# Green Synthesis of Silver Nanoparticles by Extracellular Extracts from *Aspergillus japonicus* PJ01

**DOI:** 10.3390/molecules26154479

**Published:** 2021-07-24

**Authors:** Pei-Jun Li, Jiang-Juan Pan, Li-Jun Tao, Xia Li, Dong-Lin Su, Yang Shan, Hai-Yun Li

**Affiliations:** 1 Guangxi Key Laboratory of Electrochemical and Magnetochemical Function Materials, College of Chemistry and Bioengineering, Guilin University of Technology, Guilin 541004, China; 19580766725@sohu.com (J.-J.P.); taolj@guilinpharma.com (L.-J.T.); lix754@glut.edu.cn (X.L.); sy6302@sohu.com (Y.S.); 2 Hunan Agricultural Product Processing Institute, Hunan Academy of Agricultural Sciences, Changsha 410125, China; liaolili8856@aliyun.com

**Keywords:** green synthesis, silver nanoparticles, extracellular extracts, antibacterial properties

## Abstract

The present study focuses on the biological synthesis, characterization, and antibacterial activities of silver nanoparticles (AgNPs) using extracellular extracts of *Aspergillus japonicus* PJ01.The optimal conditions of the synthesis process were: 10 mL of extracellular extracts, 1 mL of AgNO_3_ (0.8 mol/L), 4 mL of NaOH solution (1.5 mol/L), 30 °C, and a reaction time of 1 min. The characterizations of AgNPs were tested by UV-visible spectrophotometry, zeta potential, scanning electron microscope (SEM), transmission electron microscopy (TEM), X-ray diffraction (XRD), and thermogravimetric (TG) analyses. Fourier transform infrared spectroscopy (FTIR) analysis showed that Ag^+^ was reduced by the extracellular extracts, which consisted chiefly of soluble proteins and reducing sugars. In this work, AgNO_3_ concentration played an important role in the physicochemical properties and antibacterial properties of AgNPs. Under the AgNO_3_ concentration of 0.2 and 0.8 mol/L, the diameters of AgNPs were 3.8 ± 1.1 and 9.1 ± 2.9 nm, respectively. In addition, smaller-sized AgNPs showed higher antimicrobial properties, and the minimum inhibitory concentration (MIC) values against both *E. coli* and *S. aureus* were 0.32 mg/mL.

## 1. Introduction

In recent years, mental nanoparticles (MNPs) have been applied widely in many fields, such as catalysis, optics, antimicrobials, etc. Usually, chemical reduction was the most frequent method for the preparation of MNPs; however, organic solvents and toxic reducing agents might cause potential environmental and biological hazards [1]. Recently, the preparation of MNPs in an aqueous solution by using polymers as stabilizing agents has been reported. Rani et al. [2] synthesized thiourea-linked polymer as a starting entity to develop a sensor for quinalphos detection. Huerta-Aguilar et al. [3] used thiophene-based organic nanoparticles decorated with AuNPs to detect cysteine and cytosine in real samples, such as *Saccharomyces* *cerevisiae* cells.

Biogenic synthesis of AgNPs can be performed by using organisms such as bacteria, fungi, plants, or the by-products of their metabolism, which act as reducing and stabilizing agents. Among these organisms, fungi are attractive because they offer high tolerance to metals and are easy to handle. They also secrete large quantities of extracellular proteins that contribute to the stability of the nanoparticles [4]. Generally, the procedure includes the following steps: culturing fungus on agar, transferring them to a liquid medium, filtering fermentation broth, and adding silver nitrate to the filtrate [5,6]. Fungal biomass or metabolites is usually harvested in submerged fermentation (SmF). Compared to SmF, solid-state fermentation (SSF) presents unparalleled advantages, such as higher productivity per reactor volume, lower capital and operating costs, lower space requirements, simpler equipment, and easier downstream processing [7]. However, most fungal extracellular extracts used for biogenic synthesis of AgNPs were derived from SmF; few reports focused on SSF.

Among the fungal extracellular extracts, many biomolecules can be used as reducing agents and stabilizers for AgNPs biosynthesis. Nicotinamide adenine dinucleotide (NADH) and NADH-dependent nitrate reductase are considered to be the most important factor in the biogenic synthesis of metallic nanoparticles [8,9]. Mishra et al. [10] reported the synthesis of AgNPs by using neem leaf extract containing alpha amylase enzyme and fungal cellulase. Vetchinkina et al. [11] believed that the bioreduction of the metal-containing compounds and the formation rate of Au and Ag nanoparticles depended directly on the phenol oxidase activity of the fungal extracts used. Except for microbial enzymes, reducing sugars and polysaccharides are also the main components of fungal extracellular extracts, which can be used as reducing agents or stabilizers in the synthesis of metal nanoparticles. In our previous report [7], *A. japonicus* PJ01 was used for multi-enzyme complexes (MEC) production under SSF, and the MEC contained pectinase, CMCase, and xylanase. As far as we know, there were few reports on the preparation of AgNPs by the fungal multi-enzyme complexes, and the reaction mechanism remained to be solved.

In this study, we report a simple method to synthesize AgNPs by using a cell-free extracellular extracts of *A. japonicus*. The reaction process was optimized through detecting the intensity of UV-vis spectroscopy. The characterizations of AgNPs were performed by zeta potential, transmission electron microscopy (TEM), scanning electron microscope (SEM), Fourier transform infrared spectroscopy (FTIR), X-ray diffraction (XRD), and thermogravimetric (TG) analyses. The antibacterial activities of AgNPs were evaluated by disc diffusion assays and growth inhibition curve tests. This study will provide a green, efficient, and low-cost method for the preparation of AgNPs. It is of great significance for developing a new bio-based AgNPs antibacterial agent.

## 2. Results

### 2.1. Optimization of the Biological Synthesis of AgNPs

In a previous report [7], *A. japonicus* PJ01 was used for multi-enzyme complexes (MEC) production under solid-state fermentation (SSF). In the extracellular extracts, pectinase, CMCase, and xylanase activities were 48.3 ± 0.2, 2.9 ± 0.1, and 50.2 ± 0.2 U/mL, respectively. The extracellular extracts were composed of soluble proteins (0.14 ± 0.2 mg/mL), reducing sugars (0.54 ± 0.4 mg/mL), and carbohydrates (0.81 ± 0.3 mg/mL). We investigated the reaction factors of AgNPs biosynthesis, including AgNO_3_ concentration, NaOH concentration, reaction time, and temperature.

#### 2.1.1. Effect of AgNO_3_ Concentration

**Figure 1:**
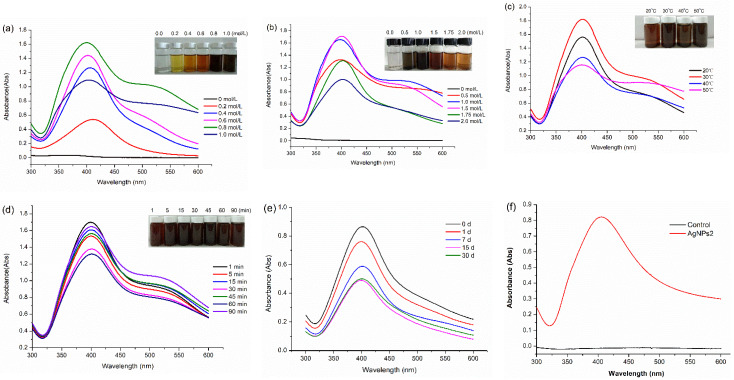
Effects of AgNO_3_ concentration (**a**), NaOH concentration (**b**), temperature (**c**), and reaction time (**d**) on AgNPs preparation; the stability of AgNPs2 (**e**); UV result of AgNPs2 and control (**f**); control was the wheat bran extract without inoculation.

According to the method of Section 4.3, 10 mL of extracellular extracts was heated to 30 °C, 1 mL of AgNO_3_ solution (0–1.0 mol/L) was added drop by drop, and then, 4 mL of NaOH solution (1.0 mol/L) was added. Then, the latter solution was placed in a magnetic stirrer at 30 °C for 1 min. Figure 1a shows the influence of AgNO_3_ concentration on the production of AgNPs. When the concentration of silver nitrate is at a relatively low level (0.2 mol/L), the absorption peak is also relatively low. As the concentration of AgNO_3_ increased to 0.8 mol/L, the peak intensity increased gradually to the maximum value. Meanwhile, the peaks became flattened, which indicated the existence of different sizes of AgNPs. In addition, the color of the AgNPs solution became darker with the increase of AgNO_3_ concentration. In the following optimal experiments, AgNO_3_ concentration was fixed at 0.8 mol/L. In order to compare the effects of AgNO_3_ concentration on the particle size of AgNPs, 0.2 and 0.8 mol/L of AgNO_3_ were selected as precursors, and the AgNPs were named as AgNPs1 and AgNPs2, respectively.

#### 2.1.2. Effect of NaOH Concentration

Figure 1b indicates that the absorbance peaks reached their maximum value when the alkali concentration increased from 0.5 to 1.5 mol/L, and then the peaks decreased gradually. The color of nano silver colloid solution gradually became deeper with NaOH concentration up to 1.0 mol/L, and after that, the color gradually became lighter. The results imply that alkaline is very important for the synthesis of AgNPs.

#### 2.1.3. Effect of Temperature

Figure 1c indicates that temperature plays an important role in the synthesis of AgNPs. When the temperature increased from 20 to 30 °C, the peak intensity reached the maximum value. After that, the peak values decreased significantly. In addition, broader peaks were observed at a high temperature, which indicated the existence of different sizes of AgNPs.

#### 2.1.4. Effect of the Reaction Time

In this work, the effect of reaction time (1–90 min) on the formation of AgNPs was not obvious (Figure 1d). When the reaction time was 1 min, the absorbance was the highest, which indicated that 1 min is enough for the formation of AgNPs. The peak values began to decrease with the increase of reaction time. This indicates that longer reaction time is not favorable for the formation of nanoparticles.

#### 2.1.5. Stability of AgNPs2

Figure 1e indicates that AgNPs2 colloids are stable at room temperature. With the prolongation of the standing time, the peak values of AgNPs2 decreased slowly. When the standing time reached 30 days, the peak value of AgNPs2 did not decrease anymore, which indicated that AgNPs2 did not agglomerate and the colloids tended to be stable. Figure 1f showed that no absorption peak was found from the UV results of the control sample (control was the wheat bran extract without inoculation), which confirmed that fermented fungi extract was essential for AgNPs synthesis.

### 2.2. Characterization of the AgNPs

#### 2.2.1. Zeta Potential and Particle Size Distribution

**Figure 2:**
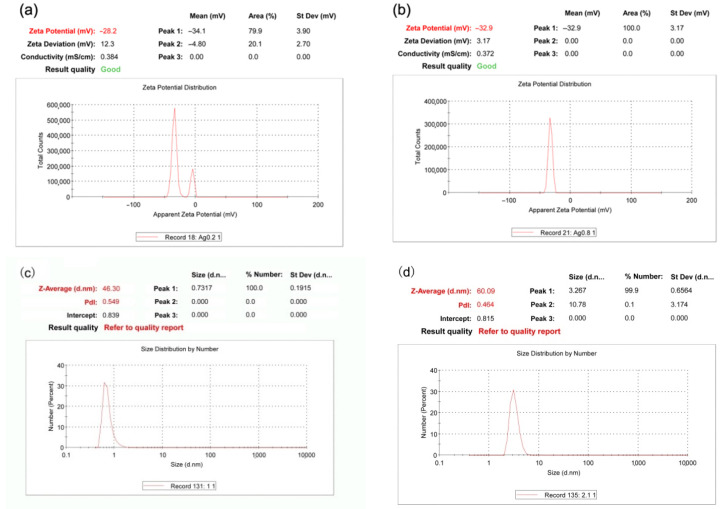
Zeta potentials and particle size distributions of AgNPs1 (**a**,**c**) and AgNPs2 (**b**,**d**). AgNPs1 and AgNPs2 were synthesized under different AgNO_3_ concentration of 0.2 and 0.8 mol/L, respectively.

Zeta potential analysis is a tool for predicting the stability of the particles suspended in colloidal solutions. The zeta potentials of AgNPs1 and 2 are −28.2 and −32.9 mV, respectively (Figure 2). These results indicate that AgNPs has negative surface charges, and AgNPs2 is more stable than AgNPs1. DLS sizing data confirmed that the majority of AgNPs created were under 10 nm in diameter; however, due to the technique’s tendency to overestimate particle size. Figure 2c,d show that the mean particle sizes of AgNPs1 and 2 are 46.30 and 60.09 nm, respectively. Polydispersity index (PdI) is a measure of particles homogeneity, and the values closer to 0.3 indicate that stable solutions of aggregates have formed.

#### 2.2.2. TEM and SEM Images

**Figure 3:**
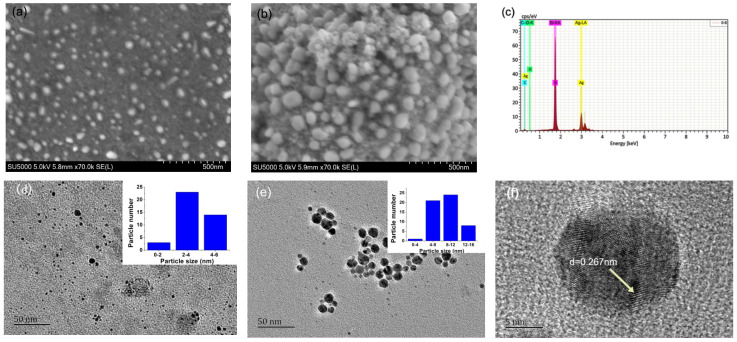
The SEM and TEM images of AgNPs1 (**a**,**d**) and AgNPs2 (**b**,**e**), EDS (**c**) and crystal (**f**) analysis of AgNPs2.

Figure 3a,b show the microstructure of the AgNPs1 and AgNPs2, respectively. Different spherical shape and irregular shape can be clearly seen from SEM images. AgNPs2 was larger than AgNPs1, which indicated that higher AgNO_3_ concentration was beneficial to the formation of larger AgNPs. Figure 1c shows that elements of AgNPs2 include Si (51.92%), Ag (43.66%), C (4.30%), and O (0.28%). Element Si comes from silicon carrier in SEM detection, and the weak signal of carbon and oxygen may be attributed to the proteins and carbohydrates capped on the AgNPs. However, N and S elements were not detected in AgNPs by the energy spectrum, which meant that the content of proteins was lower than that of carbohydrate. Figure 3d,e show the TEM images of AgNPs1 and 2, respectively. The average diameters of AgNPs1 and 2 are 3.8 ± 1.1 and 9.1 ± 2.9 nm, respectively, which are consistent with the SEM results. Figure 3f shows well-defined lattice fringes of the Ag (1 1 1) plane in AgNPs, which confirms that highly dispersed metallic AgNPs formation.

#### 2.2.3. FTIR, XRD, TGA and XPS Analysis

**Figure 4:**
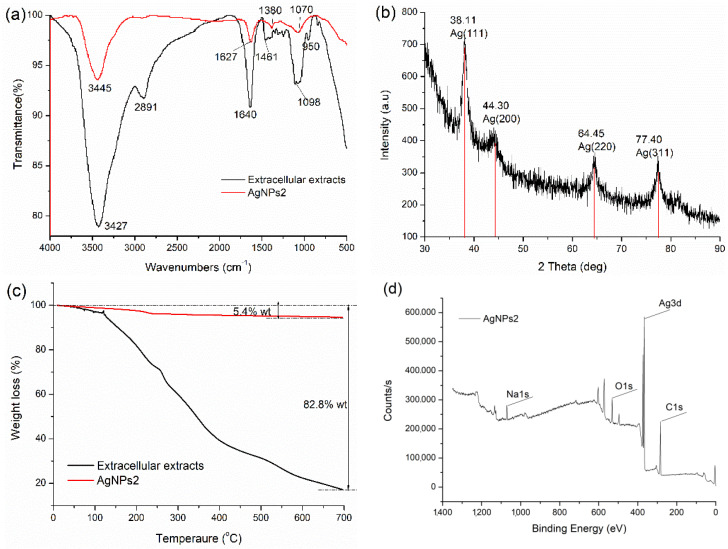
Physical–chemical characterization of AgNPs2. (**a**) FTIR spectra; (**b**) XRD spectrum; (**c**) TGA thermograms; (**d**) XPS spectra.

The typical FTIR absorption spectra of extracellular extracts and AgNPs2 are presented in Figure 4a. In extracellular extracts, the peaks corresponding to 3427, 2891, 1640, and 1098 cm^−1^ are attributed to O-H, C-H, -C=O, and C-OH, respectively [12]. In synthesized AgNPs, bands of extracts at 3427 and 1640 cm^−1^ shifted to 3445 and 1627 cm^−1^, respectively. The results indicated that the O-H and C=O functional groups were involved in the synthesis of AgNPs [13]. The gentle shift in the amide stretching peak changed from 1640 to 1627 cm^−1^. It could be attributed to the electrostatic interaction between the surface of AgNPs and the amide bonds of proteins, which was in line with the previous literature [14].

Figure 4b shows that the XRD peak positions are consistent with metallic silver. The peak values are located at 38.11°, 44.30°, 64.45°, and 77.40° (JCPDS 85-1326), which correspond to the Miller indices (111), (200), (220), and (311), respectively [13]. TGA is commonly employed to provide the information of the Ag core and stabilizer ratio of the silver nanoparticles directly [15]. The results show the slight weight loss under 150 °C, which is due to the evaporation of water. In the temperature range from 160 to 700 °C, the organic macromolecules from extracellular extracts are found to decompose and fall off from the surface of AgNPs. As can be seen from Figure 4c, the content of the organic part (thermally decomposable) in extracellular extracts and AgNP2 were 5.4% *w*/*w* and 82.8% *w*/*w*, respectively. It can be seen that Ag accounts for the vast majority of AgNPs, which is far more than the content of organic matter. Figure 4d shows the complete XPS spectra from 0 to 1300 eV of AgNPs2, which indicates that the composite contains C, Ag, O, and Na. The C and O peak is due to the biomacromolecule of the extracts.

### 2.3. Antibacterial and Antifungal Activities of AgNPs

**Figure 5:**
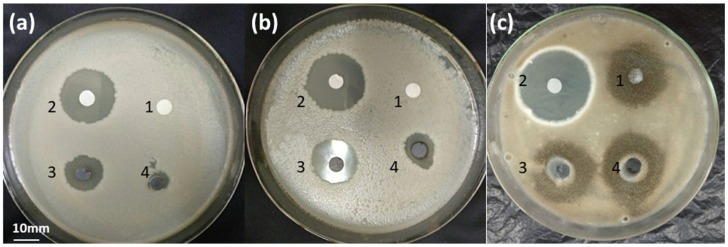
Antimicrobial activities of AgNPs against *E. coli* (**a**), *S. aureus* (**b**), and *A. japonicus* (**c**). 1, negative control (extracellular extracts); 2, positive control (**a**-2 and **b**-2 is ampicillin; **c**-2 is econazole nitrate); 3, AgNPs1; 4, AgNPs2.

The antibacterial activities of AgNPs were evaluated by disc diffusion assays and growth inhibition curve tests. Figure 5 and Table 1 show the antimicrobial activities of AgNPs by using the inhibition zones. Table 1 shows that the inhibition zones of *E. coli* and *S. aureus* caused by AgNPs1 are 13.4 ± 0.2 and 17.0 ± 0.3 mm, respectively. However, the inhibition zones from AgNPs2 are 8.2 ± 0.1 and 12.1 ± 0.2 mm, respectively. AgNPs1 showed stronger antibacterial activities against bacteria because of its smaller particle size. However, the antifungal activities of AgNPs were not obvious, which was attributed to the different antibacterial mechanisms between fungi and bacteria.

**Figure 6:**
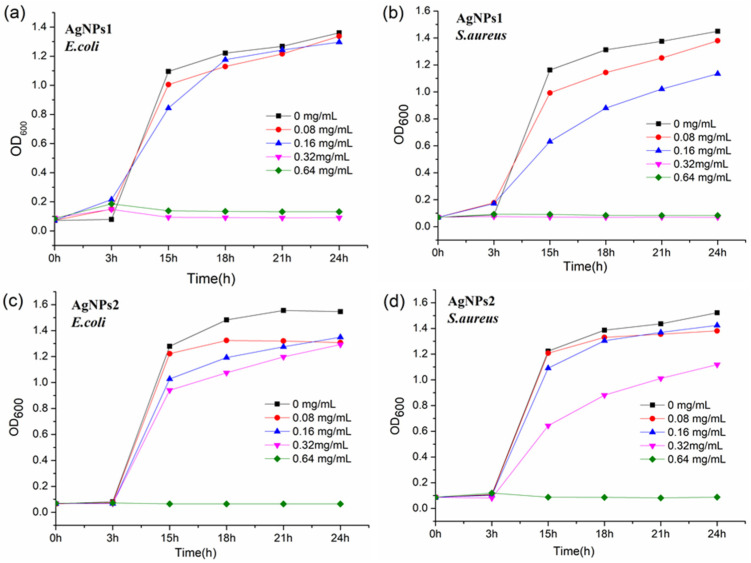
*E. coli* and *S. aureus* growth curves at different concentrations of AgNPs1 (**a**,**b**) and AgNPs2 (**c**,**d**), respectively.

Figure 6 shows the antibacterial activities of AgNPs by using the bacterial growth curve tests. The results indicate that the MICs of AgNPs1 against *E. coli* and *S. aureus* were 0.32 mg/mL (Figure 6a,b); however, the MICs of AgNPs2 against the two bacteria were 0.64 mg/mL (Figure 6c,d). All of these results reveal that smaller-sized AgNPs1 show higher antimicrobial activities, which is in accordance with the inhibition zone tests (Table 1).

## 3. Discussion

According to our previous report [7], the main organic matter in the extracellular extracts was composed of soluble proteins, reducing sugars, and polysaccharides. The soluble proteins stem from the fungal metabolites (enzymes and proteins) and wheat bran. The enzymes were mainly composed of carbohydrate hydrolases, such as pectinase, CMCase, and xylanase. On the other hand, reducing sugars might come from the degradation products of bran polysaccharide, and the polysaccharides might come from exopolysaccharides produced by *A. japonicus* PJ01. In this work, reducing sugars could be used as reducing agents, and polysaccharides and proteins could be used as stabilizing agents.

During the biological synthesis of metallic nanoparticles, a number of controllable factors were involved in the nucleation and subsequent formation of stabilized nanoparticles. These factors included pH, reactant concentrations, reaction time, and temperature [6,16]. In this work, we optimized the following reaction conditions: AgNO_3_ concentration, NaOH concentration, temperature, and reaction time. Firstly, there was a gradual increase in the absorption peak of AgNPs by changing the concentration of AgNO_3_ from 0.2 to 0.8 mol/L, and after that, the peaks began to decrease and became flat (Figure 1a). Rose et al. [17] believed that the distortion in the peak symmetry and flatten peak indicated non-uniformity in particle size. Furthermore, the size of prepared AgNPs could be controlled by AgNO_3_ concentration. In this work, with the AgNO_3_ concentrations increased from 0.2 to 0.8 mol/L, the diameters of AgNPs increased from 3.8 ± 1.1 to 9.1 ± 2.9 nm (Figure 3). The results were coincident with the previous report, which revealed that the smallest AgNPs were obtained at 10^−2^ mol/L of metal ion, and an excess addition of metal ions with a concentration 10^−1^ mol/L resulted in the formation of very large particles [18]. Secondly, pH can be used to control certain characteristics of the nanoparticles [6]. Qian et al. [19] observed that alkaline pH favored the synthesis of silver nanoparticles when AgNO_3_ was added to the filtrate of the fungus *Epicoccum nigrum*. Figure 1b shows that the absorption peaks became lower as the NaOH concentration exceeded 1.5 mol/L, which could be explained by the enzyme probably deactivating gradually, and this may be the reason for reduced synthesis at higher pH values [18]. Thirdly, temperature can affect the synthesis speed, the size, and the stability of the nanoparticles. Figure 1c shows that the optimized temperature is 30 °C, and higher temperatures lead to the decrease of absorption peaks. AbdelRahim et al. [20] explained that higher temperature led to protein inactivation, which led to larger particle size. At an increased temperature, the kinetic energy of the AgNPs in the solution also increases and collision frequency between the particles also rises, resulting in a higher rate of agglomeration [21]. In this work, the synthesis had been completed within 1 min at 30 °C, which reflected the higher synthesis efficiency and lower cost than previous reports (Figure 1d). For instance, Azmath et al. [22] found that the reaction rate increased at higher temperatures, and the synthesis was completed within 20 min at temperatures above 50 °C. AbdelRahim et al. [20] obtained the highest production of AgNPs after 48 h of incubations by using *Rhizopus stolonifer* mycelial filtrate.

In general, nanoparticles with zeta potential greater than +25 mV or less than −25 mV have sufficient electrostatic repulsion to remain stable in solution [5,23]. The zeta potentials obtained for samples AgNPs1 and 2 were −28.2 mV and −32.9 mV, respectively (Figure 2). The zeta potential values indicated good stability of the AgNPs colloids. In addition, the AgNPs solutions were monitored at time intervals of 1, 7, 15, and 30 days by using a UV–Vis spectroscope, and the absorbance peaks remained at a high level, which meant no obvious condensation and sedimentation (Figure 1e).

FTIR measurements (Figure 4a) were carried out to verify the possible interaction between the silver ions and the functional groups of biomolecules. The peak at 1380 cm^−1^ was due to the symmetrical C-O stretching of carboxyl groups [4]. Compared with fungal fermentation extracts, the band from AgNPs became much sharper and stronger (Figure 4a), which indicated that the aldehyde groups in these reducing sugars were oxidized to carboxyl groups by Ag^+^ [24]. Except for carbohydrates, the presence of proteins in *A. japonicus* PJ01 extracts may have been responsible for stabilizing the synthesized NPs. For example, the peak at 3427 cm^−1^ indicates N-H stretching vibrations in amide linkages of proteins. The band at 1640 cm^−1^, referring to the carbonyl stretch, is assigned to the amide I bond of protein. The band at 1461 cm^−1^ may be assigned to methylene scissoring vibrations from the proteins/biomolecules in the solutions, and the band at 950 cm^−1^ corresponds to an aromatic ring. The results indicate that the presence of the proteins and enzymes could reduce the Ag^+^ ions to atoms and form AgNPs [25]. In brief, the FTIR analysis showed that the reduction of Ag^+^ was a combination action of proteins and reducing sugars.

Although many studies have reported the biogenic synthesis of AgNPs using fungi, the specific mechanisms have not yet been fully elucidated [6]. It was usually considered that enzymes played a key role on AgNPs preparation from fungal fermentation extract. Chowdhury et al. [26] reported that the presence of an 85 kDa protein band was responsible for the capping and stabilization of the AgNPs by using SDS-PAGE analysis. Except for soluble proteins, there are many other microbial metabolites and fermentation media affecting the synthesis of AgNPs. For instance, the growth media of bacterial culture played an important role in the synthesis of metallic nanoparticles with regard to their size and shape [27]. In the follow-up study, the following factors will be considered, such as microbial growth medium, secreted proteins, reducing sugars, and polysaccharides. Furthermore, it is necessary to determine the composition of the extracts, which will reveal the synthesis mechanism of metallic nanoparticles.

Several studies have shown that the nanoparticles smaller than 10 nm could penetrate to the interior of bacterial cells, and this increased their bactericidal activities. While in general, nanoparticles greater than 10 nm cannot penetrate the interior of bacteria, which decreases its bactericidal power [28]. The antibacterial tests revealed that smaller-sized AgNPs1 (3.8 ± 1.1 nm) showed higher antimicrobial activities than AgNPs2 (9.1 ± 2.9 nm), which was consistent with the previous results. Furthermore, MIC results show that growth control is more effective for Gram-positive bacteria than for Gram-negative bacteria (Table 1). This is probably because the former bacteria have simpler cell membrane structures, whereas the latter possess a three-layer configuration, decreasing the permeability of the NPs into the cell membrane [29]. We propose that smaller-sized AgNPs could adsorb onto the surface of the cell membrane, penetrate the cell membrane, and hence affect cell function including replication and respiration. The simultaneous production of reactive oxygen species (ROS) from NPs as well as from Ag oxidation inside cells may affect cell functions in a shorter time, causing severe effects on the cells, and leading to rapid cell death [29]. Compared with sodium borohydride [30], AgNPs prepared by fermentation broth did not show higher antibacterial efficiency. However, the green preparation process avoids environmental pollution and shows the advantages of lower cost and higher efficiency.

Finally, compared with AgNPs, antibiotic ampicillin and econazole nitrate showed stronger antibacterial activities against bacteria and mold, respectively (Figure 5). This means that the traditional antibiotics still have relatively strong antibacterial abilities. In order to improve the antibacterial property, Sidhu et al. [31] engaged the penicillin G as a carbon source for the synthesis of penicillin carbon dots (PCDs), which made the carbon dots more aggressive toward pathogenic microbes. Therefore, adding antibiotics in the preparation process of nanoparticles might enhance their antibacterial efficiency.

## 4. Materials and Methods

### 4.1. Materials and Reagents

Silver nitrate (AgNO_3_, AR, ≥99.8%) was purchased from Xilong Scientific Co., Ltd. (Shantou, China). Ampicillin and econazole nitrate were purchased from J&K Scientific Ltd. (Beijing, China), nutrient broths for antibacterial and antifungal activities tests were purchased from Qingdao Hope Bio-Technology Co., Ltd. (Qingdao, China), and the other reagents were analytically pure.

### 4.2. Extracellular Extracts

Extracellular extracts was prepared according to previous method [7]. Briefly, *A. japonicus* PJ01 was used for solid-state fermentation at 30 °C for 72 h, and wheat bran was selected as the sole substrate. Extraction was carried out at a solid/liquid ratio of 1:20 on a rotary shaker at 170 rpm for 45 min. Extracts were filtered through coarse filter paper, filtrates were centrifuged at 10,000× *g* for 10 min at 4 °C, and the supernatants were used as extracellular extracts solutions. Reducing sugar content of extracellular extracts was determined by the DNS method. Soluble protein was determined by the modified Bradford method using bovine serum albumin (BSA) as a standard. The carbohydrates were determined by the phenol–sulfuric acid method using glucose as a standard [32].

### 4.3. Preparation of AgNPs

We performed single factor experiments by varying AgNO_3_ (0–1.0 mol/L), NaOH (0–2.0 mol/L), reaction temperature (20–50 °C), and time (0–90 min). Firstly, 10 mL of extracellular extracts was heated to set temperature, 1 mL of AgNO_3_ solution was added drop by drop, and then, 4 mL of NaOH solution was added. Then, the latter solution was placed in magnetic stirrer at fixed temperature and time. Then, the solution was cooled to room temperature. In order to obtain AgNPs powders, the solution was centrifuged for 10 min at 10,000 rpm, and the slurry was washed thrice with distilled water and dried in hot air oven (60 °C) for 10 h. The AgNPs samples prepared under different AgNPs concentration were named as AgNPs1 and 2, respectively (Table 2).

### 4.4. Characterization of AgNPs

According to previous methods [1,12], the AgNPs in colloidal solution were monitored using UV-vis spectra TU-1950 spectrophotemeter (Beijing, China), Zeta sizer Nano ZS90 (Malvern Instruments, Malvern, UK), and transmission electron microscope (TEM, JEOL-JEM-2100F, Japan), respectively. The dried AgNPs powders were subjected to FE-SEM (S-5000, Hitachi Co., Ltd., Matsuda, Japan), X-ray diffraction (XRD, PANalytical B.V., X’Pert^3^ Powder), and FTIR spectra Nicolet is10 (Thermo Fisher Scientific, Waltham, MA, USA), respectively. The thermal behavior of the prepared AgNPs was recorded with a thermogravimetric analyzer SDT Q600 (TA Instruments) in a nitrogen atmosphere at a heating rate of 10 °C/min from 25 to 700 °C. X-ray photoelectron spectroscopy (XPS, ESCALAB 250Xi, Thermo Fisher Scientific, USA) was conducted to determine the chemical states.

### 4.5. Antibacterial and Antifungal Activities

The antibacterial activities of AgNPs powders were analyzed according to previous methods [12]. Briefly, a suspension of culture was spread over the surface of agar plates with the help of a sterile cotton swab. The sterile discs (6.00 mm, Whatman No.1 filter paper) were impregnated with 100 µg AgNPs, while positive controls were ampicillin (20 µg, for bacteria) and econazole nitrate (20 µg, for fungi); then, the discs were incubated on bacteria culture plates at 37 °C for 24 h (fungi, 30 °C for 48 h) to determine the bacterial inhibition zones. MICs were visually identified as the lowest concentration of test compound that inhibited the visible growth and was confirmed by measuring the OD_600_ of all treatments. A series of dilutions were prepared to final concentrations of 0–0.64 mg/mL; then, the inoculum was mixed with LB medium and incubated at 37 °C for 24 h. The sample with no AgNPs was termed as blank control. All of the antimicrobial tests were performed in triplicate.

## 5. Conclusions

The present study reports a simple and environmental benign approach for the biosynthesis of AgNPs using extracellular extracts from solid-state fermentation of *A. japonicus* PJ01. It is probable that soluble proteins, reducing sugars, and carbohydrates are involved in the bioreduction and synthesis of AgNPs. Fungal culture filtrate mediated the successful synthesis of spherical-shaped AgNPs of 3.8 and 9.1 nm in size under different AgNO_3_ concentrations. The AgNPs exhibited effective antibacterial and antifungal activities, and smaller-sized AgNPs1 showed higher antimicrobial activities.

## Figures and Tables

**Table 1:** Diameters of inhibition zone of AgNPs against bacteria and fungi.

Bacteria and Fungi	Diameters of Inhibition Zone (mm)		
	AgNPs1	AgNPs2	Positive Control ^1^
*E. coli*	13.4 ± 0.2	8.2 ± 0.1	18.8 ± 0.2
*S. aureus*	17.0 ± 0.3	12.1 ± 0.2	22.8 ± 0.1
*A. japonicus*	8.8 ± 0.2	8.4 ± 0.2	29.2 ± 0.3

^1^ Positive control (ampicillin and econazole nitrate for bacteria and fungi, respectively).

**Table 2:** Reaction conditions for biosynthesis of AgNPs1 and AgNPs2.

Sample	NaOH (mol/L)	AgNO_3_ (mol/L)	Temperature (°C)	Time (min)
AgNPs1	1.5	0.2	30	1
AgNPs2	1.5	0.8	30	1

## Data Availability

Not applicable.

## Sample Availability

Samples of the AgNPs are available from the authors.
